# 

**Published:** 1881-07

**Authors:** 


**TERMS.—50 Cents a Year. Address the Bistoury, Elmira, N. Y.**

### SUBSCRIPTIONS RECEIVED UP TO JULY 12th, 1881.

NEW YORK—Mrs J M Preston, Dr. J Cottrell, R P Bowman, $3.50, J L Cooley $1, Seth Marvin, Dr R L Van Kleek, Dr. Wm Simpson $1, Edward Lamb, R T Turner, Dr John Brooman $4, Samuel Kenderling $2, Mrs A B Moore $3, Dr Henderson $1, T I Cable $1, Arthur Groom $2, Mrs. N B Fassett $2, Harris Dunn $3, Dr O L Simmons $3, Dr S Peters $1, Dr A N Leech $2, Dr Jas Horr $2, Dr M R May $4, Peter Holt $1, Mrs Dan Smith $1, Mrs D W Beverly $3, Mrs H H Snow $2.50, Dr O H Hunt $2, Dr Amos Smith $1.50, Dr. N N Brooks $3, Dr R Josylin $1.50, Aaron Byle 2, Samuel Gunn $3, Dr A A Folks $2.50, Dr P L Johnson $2, Dr Hamilton Squire $2.50, Dr D D Hind $2, Dr B D Howe $5.50, Louis Smith $1, H H Harris $1, Dr J D Bowers $3.50, Dr Homer Rose $4, P J Seacor $2.

PENNSYLVANIA.—R J Wisner $1, Dr P Hermann, Wm Scott, Dr D Eldridge Rice, Dr Max Harding $2, Dr H O Smith $4, Dr Peter Hunt $2, Dr A B Leisenring $2.50, Dr. Hiram Duvalle $2.50, Dr J W Cummings $2, Dr Dan Schultz $2.50, Dr J Eby $2, Dr D P Bloss $2.50, Dr J Davis $2, Dr N N Smith $2 50, Dr L B Fruct $2, Dr H R Lamb $2.50, Dr Smith Blunt $4, Dr Doun Eldrsdge $3, Dr H Atwater $2.50, Dr Smith Dix $1.50, Dr D D Dunn $6, Mrs J Able, Mrs C France $1, J R Peters, Alvin Cooper, Dr D Davis $2, Dr A L Hard $1.

DISTRICT COLUMBIA.—A Zoller, P D Mix, Mrs A D Lamb $2.

FLORIDA—Dr Sam Jones $2, Dr A B Mitchell $1.50, Mrs T D Jones $1.

GEORGIA—Dr O B Krutzman $1 50, Mrs L Wharton $1 50.

INDIANA—Dr T Faulks $1, Dr S B Jennings $1, D Daniels.

ILLINOIS—Dr Samuel Harris $1, John Wintermute.

KENTUCKY—Dr W Mayo Dr Geo Donders.

MASSACHUSETTS—Dr J Richards, Dr Calvin Snow $1, Mrs Sunderland $1 50, Dr Louis Moss $1, Dr B Summerville $1 50.

MISSOURI—Dr James Keaney, Dr John Wolf $2, P D Tisdale, Mrs A N Fox $1 50, Dr B St John $1 50.

OHIO—Dr Jno Senseman, W M Chesney, Dr J A Henshall, Dr P DeForest $2, Dr J LaSelle $1 50.

MICHIGAN—Dr W R McLaren $1, Dr A A Whitney $1.

TEXAS—I S Dial $1, Dr John Lyon $2.

TENNESSEE—Dr Theo M Gault.

WISCONSIN—Dr C Wiensma, Dr Jas Dunn, Dr N Jones $2, Mrs S S Cox $2, Dr J I Hind $1 50.

LAWRENCEVILLE, PA—No Name $2, No address; Dr John Hunter $1.

